# Robot-Assisted Radical Prostatectomy in Solid Organ Transplant Recipients: Initial Experience and Systematic Review

**DOI:** 10.3390/cancers18152408

**Published:** 2026-07-26

**Authors:** Wojciech Połom, Sławomir Lizakowski, Katarzyna Skrobisz, Marcin Matuszewski

**Affiliations:** 1 Department of Urology, Medical University of Gdańsk, 80-214 Gdańsk, Poland; 2 Department of Nephrology, Transplantology and Internal Medicine, Medical University of Gdańsk, 80-214 Gdańsk, Poland; 3 Department of Radiology, Medical University of Gdańsk, 80-214 Gdańsk, Poland

**Keywords:** prostate cancer, robot-assisted radical prostatectomy, solid organ transplant, renal transplant, liver transplant, indocyanine green, near-infrared fluorescence, CMR Versius, ureteral identification, fluorescence-guided surgery

## Abstract

**Background/Objectives:** Prostate cancer is one of the most common non-skin solid malignancies among male solid organ transplant recipients (SOTRs), in whom radical prostatectomy is technically demanding. We report the first use of indocyanine green (ICG) fluorescence for simultaneous transplanted-ureter identification and renal graft vascular mapping during robot-assisted radical prostatectomy (RARP), and the first use of the CMR Versius^®^ platform in a SOTR. **Methods:** Retrospective case series of four consecutive male SOTRs (two renal [RTRs], two hepatic) undergoing RARP. In both RTRs, a dual-route ICG protocol was used on the da Vinci Xi with Firefly^®^ imaging: pre-docking intraureteral ICG via a ureteral catheter for ureter identification, plus an intravenous ICG bolus for graft vascular mapping and cortical perfusion. One hepatic recipient was operated with the CMR Versius^®^ system using an infra-umbilical port configuration to avoid the chevron transplant scar. **Results:** All four procedures were completed robotically without conversion. Median operative time was 176 min and median estimated blood loss 350 mL. Surgical margins were negative (R0) in all four; final pathology was pT3aN0 in three and pT2N0 in one, although in the renal recipients nodal staging reflected a contralateral-only dissection. PSA was undetectable at three months in all patients. One hepatic recipient later developed biochemical recurrence, managed with salvage radiotherapy and androgen deprivation therapy, with subsequent undetectable PSA. One hepatic recipient had a Clavien–Dindo IIIa complication. No graft dysfunction occurred. **Conclusions:** ICG-guided RARP and the CMR Versius^®^ platform appear technically feasible in carefully selected solid organ transplant recipients treated at an experienced multidisciplinary centre, with no graft-related complications observed in this small initial series. These preliminary findings require validation in larger, multicenter studies before general safety and oncological efficacy can be established.

## Simple Summary

Prostate cancer is one of the most common non-skin cancers in men who have received an organ transplant. Operating on these patients is difficult, because the transplanted kidney and its ureter, or scarring from previous liver surgery, lie close to the area the surgeon must work in, and injuring the transplanted ureter can threaten the graft. In this study we describe four transplant recipients (two kidney, two liver) who underwent robot-assisted removal of the prostate. In the kidney recipients we used a fluorescent dye that glows under near-infrared light to see the transplanted ureter and check the blood supply to the graft in real time. All four operations were completed without conversion to open surgery, the surgical margins were free of tumour on pathological examination, and no transplant was injured. Our early experience suggests that this approach is technically feasible in carefully selected transplant recipients; larger studies with longer follow-up are needed before its safety and cancer-control outcomes can be established.

## 1. Introduction

Prostate cancer (PCa) is the second most common malignancy in men worldwide and represents the most frequently diagnosed non-skin solid neoplasm among solid organ transplant recipients (SOTRs) [1,2]. Among renal transplant recipients (RTRs), PCa incidence ranges from 0.3% to 3.24% in institutional series, and genitourinary malignancies represent one of the most common non-skin solid neoplasms in this population [1]. The relationship between immunosuppression and prostate carcinogenesis remains debated: most large registry-based analyses have not demonstrated a statistically significant increase in PCa risk in solid organ transplant recipients compared to the general population, although some institutional and registry-based series report elevated standardized incidence ratios [1]. Immunosuppressive agents including azathioprine and calcineurin inhibitors have been associated with enhanced in vitro and in vivo PCa aggressiveness, although randomized data are lacking. Importantly, as transplant outcomes continue to improve and recipients live longer, the impact of cancer after transplantation has grown substantially, and malignancy is now among the most common causes of death beyond five years post-transplantation [1,2].

A clinically relevant challenge in this population is the interpretation of PSA as a screening tool. mTOR inhibitors such as sirolimus, used in only a minority of immunosuppressive regimens, have been associated with lower PSA levels than calcineurin inhibitor–based regimens in observational studies; in male renal transplant recipients, sirolimus has been reported to lower PSA by approximately 50% relative to tacrolimus-based regimens (mean 0.9 vs. 1.9 ng/mL, *p* < 0.001) [3]. This pharmacological PSA suppression may mask clinically significant disease, contributing to diagnostic delay. These observations underscore the need for lower PSA thresholds for biopsy referral and routine integration of multiparametric MRI (mpMRI) in PSA screening protocols for this population [1,2].

With the ongoing expansion of transplant eligibility criteria to older recipients, the number of male SOTRs subsequently diagnosed with PCa is increasing. In the largest contemporary multicenter cohort of RTRs with PCa, radical prostatectomy was the most frequent management modality, with 5-year biochemical recurrence-free survival broadly comparable to the general population [2]. These oncological results support an active surgical approach in appropriately selected patients [1,2,4,5].

Robot-assisted radical prostatectomy (RARP) has become the preferred surgical modality for localized PCa, offering superior visualization, precision, and minimally invasive access [4,5]. However, performing RARP in SOTRs presents unique and formidable anatomical challenges [4,6,7]. In RTRs, the transplanted kidney is typically located in the iliac fossa—most commonly the right—with its ureter coursing in close proximity to the surgical field of prostatectomy. The transplanted ureter follows an aberrant, shortened path directly to the bladder dome, lacks the periureteral fat planes present in native ureters, and is often embedded in fibrotic and adhesive tissue resulting from prior retroperitoneal surgery [4,5]. Failure to identify and protect this structure intraoperatively may result in ureteral injury with devastating consequences for graft function. In liver transplant recipients, while the upper urinary tract anatomy is preserved, the altered abdominal wall compliance, prior laparotomy, and extensive pelvic adhesions pose distinct technical obstacles.

Indocyanine green (ICG) is a water-soluble fluorescent dye that, when excited by near-infrared (NIR) light, emits a detectable fluorescent signal. Since 2010, fluorescence imaging has been integrated into the da Vinci^®^ robotic system via Firefly^®^ technology, allowing surgeons to switch in real time between conventional and near-infrared imaging modes without interrupting the procedure. In urological robotic surgery, ICG has been applied across a broad spectrum of indications, including robotic partial nephrectomy, radical prostatectomy with lymphadenectomy, ureteral reimplantation and reconstruction, and sentinel lymph node mapping [8,9,10]. In the context of ureteral identification specifically, intraureteral ICG injection under NIRF enables real-time delineation of the ureter throughout complex pelvic procedures [8,10,11,12]. Intravenous ICG has also been used to assess graft perfusion during kidney transplantation, with quantitative fluorescence angiography shown to predict early graft function [13].

Despite the growing number of SOTRs requiring surgical treatment for PCa, the published literature on RARP in this population remains limited, consisting primarily of small institutional series focused on RTRs [1,4,5,6,7]. Data on RARP in hepatic transplant recipients are extremely scarce, limited to a single case report, and—to our knowledge—no study has reported the intraoperative use of ICG fluorescence guidance specifically to identify the transplanted ureter, visualize graft vasculature, and assess renal graft perfusion during RARP. This technical gap is clinically significant: the absence of real-time anatomical guidance in this setting represents a preventable risk to graft viability.

In this case series, we report our initial experience with ICG-guided RARP on the da Vinci Xi robotic platform in four SOTRs—two renal and two hepatic transplant recipients—describing our novel fluorescence-guided technique for transplanted ureter identification and graft vascular assessment, and reviewing the current literature in the context of this unique surgical scenario.

## 2. Materials and Methods

### 2.1. Study Design and Ethics

This study combines a retrospective case series of four consecutive solid organ transplant recipients who underwent RARP at our institution, with a systematic review of the published literature on RARP in solid organ transplant recipients. The case series component was conducted in accordance with the Declaration of Helsinki. All patients provided written informed consent for the use of anonymized clinical data for research and publication purposes. This study follows the PRISMA 2020 guidelines for the systematic review component [14].

### 2.2. Systematic Review—Search Strategy and Eligibility Criteria

A systematic literature search was conducted using the PubMed/MEDLINE and Web of Science Core Collection databases. The following search terms were used in combination: (“robot-assisted radical prostatectomy” OR “RARP” OR “robotic prostatectomy” OR “laparoscopic radical prostatectomy”) AND (“renal transplant” OR “kidney transplant” OR “liver transplant” OR “hepatic transplant” OR “solid organ transplant”) AND (“prostate cancer” OR “prostate neoplasm”). No language or date restrictions were applied. All databases were last searched on 13 July 2026. Reference lists of identified articles and relevant systematic reviews were manually screened for additional eligible studies.

Inclusion criteria: primary studies reporting outcomes of RARP in patients with prior solid organ transplantation; reporting at least one of the following outcomes: operative time, estimated blood loss, surgical margin status, pathological stage, or graft function; peer-reviewed full-text articles. Exclusion criteria: single-patient case reports (*n* = 1) not providing complete perioperative and oncological outcome data; narrative review articles without original patient data (secondary systematic reviews were retained in the evidence table for contextual reference only, as their patient populations overlap with those of the included primary studies); studies reporting only open or conventional laparoscopic non-robotic prostatectomy; conference abstracts without full-text publications; duplicate patient cohorts. Two reviewers independently screened titles and abstracts; discrepancies were resolved by consensus. Single-patient case reports were not excluded on the basis of sample size; they were eligible whenever complete perioperative and oncological outcome data were reported, and were excluded only when such data were lacking, in order to minimise incomplete-reporting bias. Overlapping cohorts were identified by comparing authorship, institution and recruitment period, and only the most complete report was retained as a primary study. The review was not prospectively registered. The search was last updated on 13 July 2026; full database-specific search strategies are provided in the Supplementary Materials (Table S4). Methodological quality was independently appraised by two reviewers using the Joanna Briggs Institute (JBI) Critical Appraisal Checklists for Case Series and Case Reports [15], and, for the single matched cohort study, the JBI Checklist for Cohort Studies; disagreements were resolved by consensus. Because the evidence base consists almost exclusively of retrospective, single-arm case series, the overall certainty of evidence is low (Supplementary Table S2). The completed PRISMA 2020 checklist is provided in the Supplementary Materials (Table S1).

### 2.3. Patient Selection and Preoperative Workup

Patients were eligible for RARP if they had biopsy-confirmed localized or locally advanced prostate cancer, adequate performance status, and preserved graft function deemed compatible with general anesthesia and pneumoperitoneum. All cases were discussed within a multidisciplinary team (MDT) including urological oncology, transplant surgery, nephrology or hepatology, and anesthesiology prior to surgical planning.

Preoperative workup included serum PSA, multiparametric MRI (mpMRI) of the prostate, and contrast-enhanced CT of the abdomen and pelvis in all patients. The CT was specifically reviewed to map the position of the transplanted organ, course of the transplanted ureter (in RTRs), and graft vascular anatomy in relation to the planned operative field. Bone scintigraphy was performed selectively based on oncological risk stratification per EAU guidelines [16].

### 2.4. Surgical Platforms

Two robotic platforms were utilized across the series:

- da Vinci Xi (Intuitive Surgical, Sunnyvale, CA, USA) with integrated Firefly^®^ near-infrared fluorescence (NIRF) imaging—used in three patients (both RTRs and one hepatic transplant recipient).
- CMR Versius^®^ (CMR Surgical, Cambridge, UK)—used in one hepatic transplant recipient, selected for its inherent flexibility in trocar placement, which allowed infra-umbilical port configuration to avoid the transplant laparotomy scar. The CMR Versius^®^ platform had been previously adopted at our institution for RARP, with an initial series of 58 consecutive cases demonstrating technical feasibility, reproducible setup, and safe outcomes [17].

### 2.5. ICG Protocol

In both RTRs, indocyanine green (ICG) was administered intraoperatively via a dual-route protocol as described in the Surgical Technique section:

1. Intravenous bolus (2.5 mg in 10 mL sterile water) administered during peritoneal dissection on the side of the transplanted kidney for real-time graft vascular mapping and cortical perfusion assessment.
2. Intraureteral instillation (25 mg in 10 mL sterile water) delivered via a ureteral catheter placed endoscopically prior to robotic docking for identification of the transplanted ureter during pelvic dissection. Catheterisation of a transplanted ureter is technically more demanding than of a native ureter, owing to the angle and site of neo-implantation; in one recipient this required careful cystoscopic manoeuvring before the catheter could be advanced. A 5 Fr open-ended ureteral catheter was used and was removed intraoperatively once the surgeon had confirmed identification of the transplant ureter, rather than being retained for the duration of the procedure. All patients were screened preoperatively for contraindications to ICG (iodine or shellfish allergy, hyperthyroidism), and were monitored intraoperatively and postoperatively for ICG-related adverse events; none occurred in this series.

ICG was not employed in hepatic transplant recipients, as native ureteral anatomy was preserved in this subgroup.

### 2.6. Perioperative Management and Immunosuppression

Immunosuppressive therapy was continued throughout the perioperative period without interruption, following institutional transplant team guidance. Perioperative monitoring was standardized and focused on graft function assessment. In renal transplant recipients, serum creatinine and eGFR were measured, while in liver transplant recipients, liver function parameters (bilirubin, ALT, AST, INR) were evaluated at baseline, POD 1, 3, and 5, and at discharge. Tacrolimus trough levels were monitored on POD 1, 3, and 5 to ensure therapeutic exposure. None of the patients received an mTOR inhibitor (sirolimus or everolimus); accordingly, no PSA value reported in this series was subject to mTOR-related pharmacological suppression.

### 2.7. Outcome Measures

The following outcomes were recorded: intraoperative (operative time, estimated blood loss, intraoperative complications, conversion); pathological (final pT stage, Gleason score/Grade Group, surgical margin status R0/R1); postoperative (length of stay, Clavien-Dindo complications [18], 30-day readmission); oncological (PSA at follow-up, biochemical recurrence defined as PSA ≥ 0.2 ng/mL); and graft function (creatinine/eGFR or liver function tests at discharge and follow-up vs. baseline). In renal transplant recipients, pelvic lymph node dissection was not performed on the ipsilateral side of the graft due to dense fibrotic adhesions around the iliac vessels at the transplantation site and the risk of graft vascular injury; lymphadenectomy was limited to the contralateral side. Pelvic lymph node dissection was indicated on the basis of an estimated risk of lymph node involvement exceeding the guideline-recommended threshold, calculated using the Briganti 2012 nomogram [19] (EAU-recommended cut-off ≥ 5% [16]), consistent with the intermediate-risk disease of all four patients. In both renal transplant recipients the graft lay in the right iliac fossa, and dissection was therefore confined to the contralateral (left) obturator template; graft-side dissection was intentionally omitted to avoid injury to the transplant vasculature and the transplanted ureter. In the two hepatic recipients, bilateral obturator dissection was performed. Node yield was correspondingly low: seven nodes in total across the series (2, 2, 2 and 1 per patient), all negative, and all four patients were staged pN0 (Table 1). It must be emphasised that in the renal recipients pN0 status reflects a contralateral-only dissection and does not exclude nodal involvement on the graft side. This represents a deliberate oncological trade-off, in which the risk of nodal understaging is accepted in order to protect the graft; it should not be regarded as equivalent to a standard bilateral staging lymphadenectomy. Per-patient templates, laterality, node counts and pN status are reported in Supplementary Table S3.

## 3. Surgical Technique

### 3.1. General Operative Setup and Patient Positioning

All procedures were performed using the da Vinci Xi robotic platform (Intuitive Surgical, Sunnyvale, CA, USA) with integrated Firefly^®^ near-infrared fluorescence (NIRF) imaging, or the CMR Versius^®^ robotic system (CMR Surgical, Cambridge, UK), as detailed below. Patients were placed in the supine Trendelenburg position (25°). A transperitoneal approach was employed in all cases. Given the individual variability in transplant location, prior surgical history, and resultant adhesion patterns, trocar placement was tailored on a case-by-case basis rather than following a standardized configuration. Preoperative imaging—including contrast-enhanced CT and/or MRI of the pelvis and abdomen—was reviewed in all cases to delineate the precise anatomical relationship between the transplanted organ, its vasculature, the aberrant ureteral course, and the prostate. This planning step was considered mandatory prior to undertaking RARP in solid organ transplant recipients. In both renal transplant recipients, introduction of the 12 mm assistant trocar in its standard position was considered hazardous due to the proximity of the transplanted kidney in the iliac fossa. Under direct visual control, it was therefore placed more caudally—towards the lower abdomen—to avoid any risk of graft injury, a modification confirmed intraoperatively as necessary in each case.

Despite preoperative concerns regarding potential intraperitoneal adhesion formation secondary to prior transplant surgery, no significant adhesion burden was encountered in the operative field in any of the four cases. In the renal transplant recipients, the transplanted kidney occupies an extraperitoneal position in the iliac fossa and therefore did not contribute to intraperitoneal adhesions; the peritoneal cavity was entered cleanly and the prostate was accessible without the need for adhesiolysis in the pelvic operative field. In the hepatic transplant recipients, the liver graft is positioned in the upper abdomen and was sufficiently remote from the pelvic operative field to pose no direct anatomical obstacle during the prostatectomy.

### 3.2. RARP in Renal Transplant Recipients—ICG-Guided Technique

#### 3.2.1. Preparatory Step: Pre-Docking Cystoscopy and Ureteral Catheterization

Prior to robotic docking, with the patient already positioned and prepared for surgery, rigid cystoscopy was performed as a dedicated preparatory step. Under direct endoscopic visualization, a ureteral catheter was advanced selectively into the transplanted ureter and secured in position. In one of the two renal transplant recipients, ureteral catheterization proved technically challenging due to an unfavourable angle of the transplanted ureteral orifice at the bladder dome, requiring careful manipulation and repositioning of the cystoscope before successful catheter placement was achieved. The catheter was left in situ throughout the docking and port placement phase, serving as a conduit for subsequent intraoperative ICG instillation at the surgeon’s discretion. This pre-docking approach was deliberately chosen to avoid the need for intraoperative interruption of the robotic procedure and to ensure the catheter was reliably positioned before the operative field became inaccessible.

#### 3.2.2. Intraoperative ICG Administration—Dual-Route Protocol

**Figure 1:**
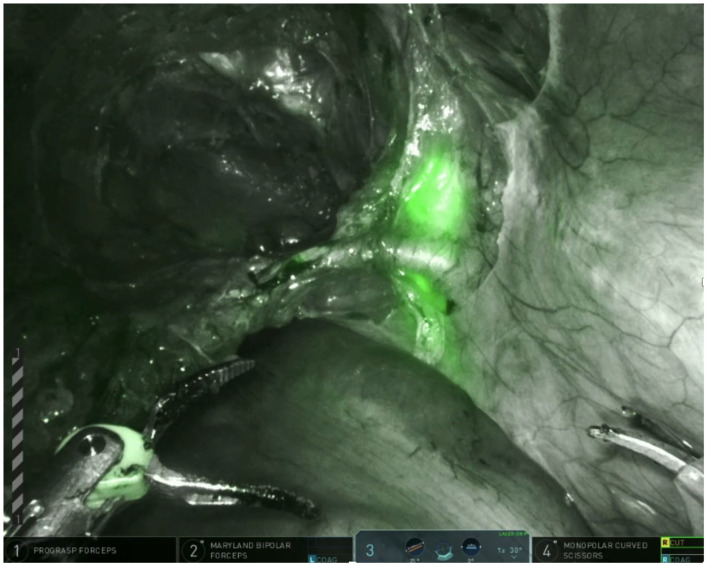
Intraoperative Firefly^®^ near-infrared fluorescence (NIRF) imaging following intravenous ICG bolus (2.5 mg) in a renal transplant recipient. The vascular pedicle of the transplanted kidney—including the renal artery and vein anastomosed to the iliac vessels—is delineated by bright green fluorescent enhancement, enabling real-time graft vascular mapping and definition of a safe dissection corridor during robot-assisted radical prostatectomy. Da Vinci Xi platform, Firefly^®^ imaging mode.

Following robotic docking, abdominal insufflation, and initial peritoneal inspection, ICG was administered via two simultaneous and complementary routes during the surgical approach to the prostate:

Intravenous bolus—graft vascular mapping and perfusion assessment. As the peritoneum overlying the iliac fossa was being opened on the side of the transplanted kidney, an intravenous bolus of ICG (2.5 mg in 10 mL sterile water) was administered by the anesthesiologist. Firefly^®^ NIRF mode was activated immediately thereafter. Within 30–60 s, the vascular pedicle of the transplanted kidney—including the renal artery and vein anastomosed to the iliac vessels—was clearly delineated by fluorescent enhancement, allowing real-time mapping of the graft vasculature and definition of a safe dissection corridor. Simultaneously, NIRF imaging provided qualitative assessment of renal cortical perfusion, confirming preserved graft uptake prior to proceeding (Figure 1). Fluorescence provided immediate and durable ureteric delineation and real-time confirmation of graft perfusion, and was maintained throughout bladder takedown—the step at which the transplanted ureter is most at risk of injury.

**Figure 2:**
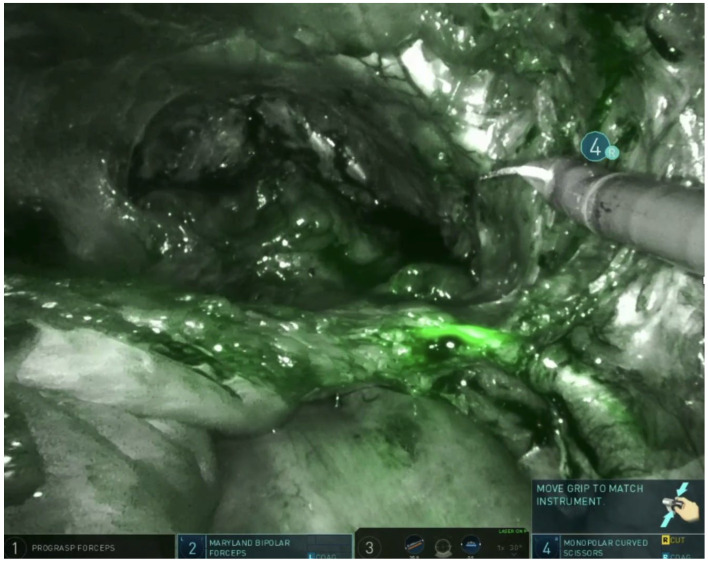
Intraoperative Firefly^®^ near-infrared fluorescence (NIRF) imaging following intraureteral ICG instillation (25 mg in 10 mL sterile water) via a pre-placed ureteral catheter in a renal transplant recipient. The transplanted ureter is visualized as a brightly fluorescent tubular structure (green), sharply contrasting with the surrounding fibrotic and adhesive pelvic tissue, enabling precise identification and protection of the transplanted ureter throughout mobilization of the bladder dome. Da Vinci Xi platform, Firefly^®^ imaging mode.

Intraureteral instillation—transplanted ureter identification. At the same operative stage—during the surgical approach and pelvic dissection—ICG solution (25 mg in 10 mL sterile water) was instilled through the previously placed ureteral catheter. Upon activation of Firefly^®^ NIRF mode, the transplanted ureter was immediately visualized as a brightly fluorescent tubular structure, sharply contrasting with the surrounding fibrotic tissue. This allowed precise delineation of the ureteral course throughout its aberrant pelvic trajectory, enabling safe mobilization of the bladder dome—where the transplanted ureter inserts—and posterior plane development without risk of ureteral injury (Figure 2).

The simultaneous availability of both vascular and ureteral fluorescent signals at the critical dissection phase constituted the key technical advantage of this dual-route ICG protocol. It is important to emphasize that ICG and Firefly^®^ NIRF imaging were employed exclusively in renal transplant recipients, in whom the aberrant course of the transplanted ureter and proximity of the graft vasculature to the operative field create a specific and well-defined intraoperative risk. In hepatic transplant recipients, where native ureteral anatomy is preserved, neither ICG nor Firefly^®^ imaging was utilized.

#### 3.2.3. Prostatectomy and Urethrovesical Anastomosis

Following safe identification and protection of the transplanted ureter and graft vasculature, the remainder of the prostatectomy proceeded according to established RARP principles: anterior and posterior bladder neck dissection, seminal vesicle dissection, development of the posterior plane (Denonvilliers’ fascia), and apical dissection with urethral transection. The urethrovesical anastomosis was constructed using a running 3-0 barbed suture (V-Loc™, Medtronic, Minneapolis, MN, USA) according to the Van Velthoven technique.

### 3.3. RARP in Hepatic Transplant Recipients—Versius Platform and Technical Considerations

In patients with prior orthotopic liver transplantation, the upper urinary tract anatomy was preserved and no fluorescence-guided ureteral identification was required. However, this group presented distinct and substantial technical challenges specific to their prior surgical history.

One of the two hepatic transplant recipients underwent RARP using the CMR Versius^®^ robotic surgical system. A key characteristic of this platform relevant to this case is its inherent flexibility in trocar placement, which allows port positions to be adjusted freely according to individual patient anatomy—a recognized feature that distinguishes it from conventional integrated-cart robotic systems.

This anatomical flexibility proved critical in the context of prior liver transplantation. The patient had a well-healed chevron (bilateral subcostal) laparotomy scar from the orthotopic liver transplantation. Standard umbilical or supraumbilical trocar placement, as employed with the da Vinci Xi system, would have necessitated either traversing scar tissue with associated risk of port-site complications, inadvertent enterotomy, or inadequate peritoneal access. Leveraging the Versius system’s inherent flexibility in port configuration, all trocars were placed below the level of the scar, entirely avoiding it. This infra-umbilical port arrangement provided safe peritoneal entry, adequate triangulation for pelvic dissection, and unobstructed BSU movement without instrument arm clashing—while maintaining the surgical geometry required for effective RARP. The CMR Versius^®^ platform had been previously adopted at our institution for standard RARP, with an initial series of 58 consecutive cases demonstrating feasibility and reproducible outcomes [17].

Following safe peritoneal access, RARP proceeded using standard transperitoneal technique. The pelvic operative field was accessible without significant adhesion burden, as the liver graft—positioned in the upper abdomen—was remote from the field of dissection. Particular attention was paid to hemostasis throughout, given the potential for subclinical coagulopathy in the context of hepatic transplantation and ongoing immunosuppressive therapy.

The second hepatic transplant recipient underwent RARP using the da Vinci Xi platform. Given the presence of the chevron transplant scar, trocars were deliberately placed more caudally than in the standard configuration, shifting port positions towards the lower abdomen to avoid scar tissue and minimize the risk of port-site complications. No intraoperative difficulties related to this trocar modification were encountered, and the surgical field was adequately exposed throughout the procedure. Taken together, the experience with both hepatic transplant recipients demonstrates that satisfactory port configuration can be achieved with either the CMR Versius^®^ or the da Vinci Xi platform in this setting, provided that trocar placement is planned individually with reference to the patient’s specific scar anatomy.

## 4. Results

All four procedures were completed robotically without conversion to open surgery. Detailed operative, pathological, and follow-up data are presented in Table 2.

### 4.1. Operative Outcomes

Median operative time was 176 min (range 150–206 min) and median console time was 150 min (range 135–165 min). Median estimated blood loss (EBL) was 350 mL (range 200–800 mL); excluding the patient with a postoperative IIIa complication requiring embolization, median EBL was 300 mL (range 200–400 mL). The highest EBL (800 mL) occurred in one hepatic transplant recipient (Patient 3, da Vinci Xi). In this patient, postoperative imaging identified a bleeding point at the site of the assistant trocar insertion, likely related to enhanced abdominal wall collateral circulation secondary to the prior hepatic transplantation and associated hepatic vascular disease. The bleeding was managed successfully by percutaneous angiographic embolization (Clavien-Dindo grade IIIa) without the need for reoperation. This complication highlights the particular risk of abdominal wall bleeding in hepatic transplant recipients, in whom portal hypertension and altered hepatic haemodynamics may give rise to prominent collateral vessels at sites of trocar insertion. No other intraoperative complications were recorded. There were no conversions to open surgery in any case.

### 4.2. ICG Findings in Renal Transplant Recipients

In both RTRs, the dual-route ICG protocol was successfully executed. Following intravenous bolus administration, the transplanted kidney’s vascular pedicle—including the anastomosed renal artery and vein—was delineated under Firefly^®^ NIRF imaging within 30–60 s, with clearly visible cortical perfusion confirming graft viability. Following intraureteral ICG instillation via the pre-placed ureteral catheter, the transplanted ureter was immediately and unambiguously identified as a fluorescent tubular structure throughout its pelvic course. No ureteral injuries occurred in either case. Graft function at discharge was preserved in both patients, with serum creatinine comparable to preoperative baseline.

### 4.3. Pathological Outcomes

Surgical margins were negative (R0) in all four patients. Final pathology was pT3aN0 in three patients and pT2N0 in one (Patient 3); clinical-to-pathological upstaging was observed in the three pT3a cases. Gleason score changes between biopsy and final pathology are detailed in Table 2.

### 4.4. Postoperative Outcomes and Follow-Up

Detailed operative, pathological, and follow-up data are presented in Table 2. No anastomotic strictures occurred. Mild stress urinary incontinence was present in three patients at 3 months; one patient was fully continent. One hepatic transplant recipient (Patient 3, operated with the CMR Versius^®^ platform) developed biochemical recurrence 4 months postoperatively (PSA 0.23 ng/mL) and underwent salvage radiotherapy to the prostate bed with androgen deprivation therapy, according to EAU-recommended schedules [16], with a subsequent decline in PSA to 0.02 ng/mL. Across the series the median follow-up was 12 months (range 4–39). At last assessment, the three patients without recurrence had undetectable PSA values (0.006, 0.003 and 0.007 ng/mL, respectively). The remaining three patients showed no biochemical recurrence and required no adjuvant or salvage therapy. Follow-up was conducted according to our standard institutional PSA-based schedule; individual follow-up intervals were not recorded prospectively, which we acknowledge as a limitation. Continence was defined as complete freedom from pad use (0 pads), and all four patients were pad-free at last assessment. The single patient with biochemical recurrence—a hepatic recipient with pT3a disease—was treated with standard salvage radiotherapy to the prostate bed together with androgen deprivation therapy. Erectile function was not assessed with a validated instrument such as the IIEF, and potency outcomes are therefore not reported. All four patients had organ-confined disease on preoperative clinical staging (cT2a–cT2b) and were of an age (49–64 years) at which preservation of erectile function was a relevant goal; bilateral nerve-sparing was therefore undertaken in every patient. Nerve-sparing status is reported per patient in Supplementary Table S3.

## 5. Systematic Review of the Literature

### 5.1. Search Results and Study Selection

**Figure 3:**
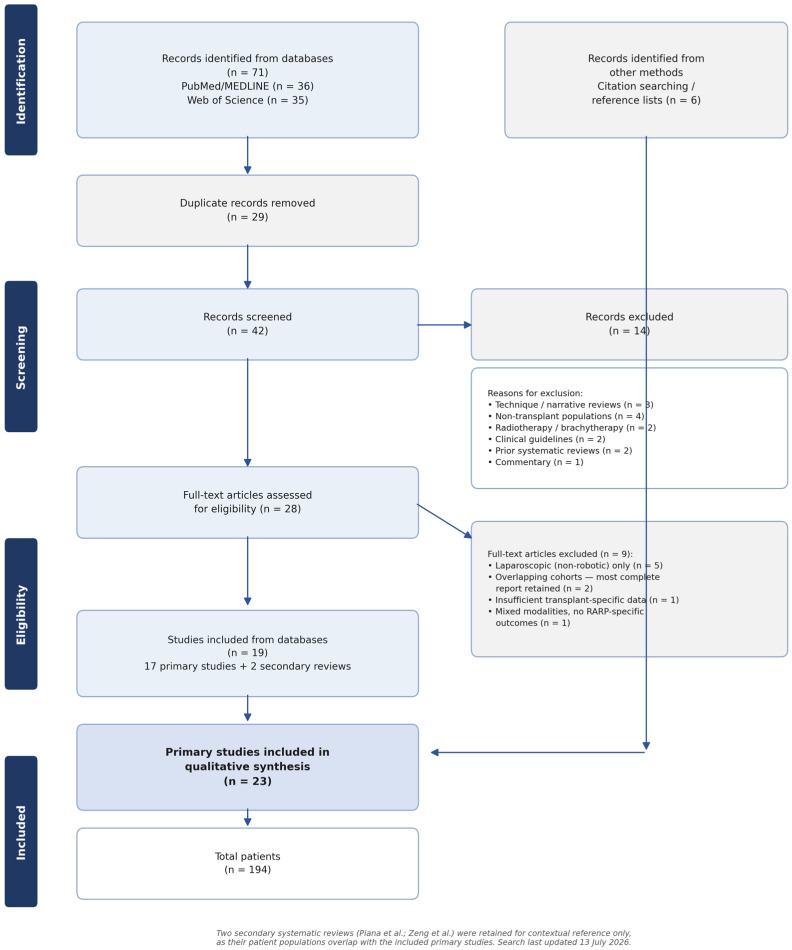
PRISMA 2020 flow diagram of study identification, screening, and selection for the systematic review of robot-assisted radical prostatectomy in solid organ transplant recipients. The systematic review was conducted and reported in accordance with the PRISMA 2020 statement [14].

The systematic database search, last updated on 13 July 2026, retrieved a total of 71 records (PubMed/MEDLINE: 36; Web of Science Core Collection: 35). A further 6 records were identified through citation searching of the reference lists of included articles and of relevant systematic reviews. After removal of 29 duplicates, 42 records remained for title and abstract screening. Of these, 14 were excluded as clearly irrelevant (3 technique or narrative reviews; 4 studies in non-transplant populations; 2 studies of radiotherapy or brachytherapy rather than surgery; 2 clinical guidelines; 2 prior systematic reviews without original patient data; and 1 commentary). Twenty-eight full-text articles were assessed for eligibility. After full-text review, 9 were excluded: 5 reporting laparoscopic (non-robotic) prostatectomy only; 2 overlapping cohorts from the same institution, for which only the most complete report was retained; 1 providing insufficient transplant-specific data; and 1 reporting mixed treatment modalities without RARP-specific outcomes. Nineteen reports from the database search met the inclusion criteria, comprising 17 primary studies and 2 secondary systematic reviews (Piana et al. [5] and Zeng et al. [20]), the latter retained for contextual reference only because their patient populations overlap with the included primary studies. Together with the 6 studies identified through citation searching, a total of 23 primary studies were included in the qualitative synthesis. The study identification and selection process is summarized in Figure 3.

### 5.2. Key Findings

Across all 23 published primary studies, comprising 194 patients in total (a denominator that excludes the four patients of the present series, which are reported separately), no intraoperative graft injury was reported. Twenty-two of the 23 primary studies enrolled renal transplant recipients exclusively. Published data on RARP in hepatic transplant recipients are limited to a single case report (Lim et al. [21]), and no series of hepatic recipients has previously been described; the present report therefore adds the largest reported experience of RARP in this population and the first to include more than one hepatic recipient. The series ranged from single-patient reports to multicenter cohorts of up to 41 patients [4,22,23]. No prior study reported the use of ICG fluorescence guidance during RARP in a solid organ transplant recipient, nor the use of the CMR Versius^®^ platform. Positive surgical margin rates ranged from 0% to 40% across studies; operative time ranged from 108 to 400 min. These findings are summarised in Table 3. Transplant-specific perioperative data for all four patients are reported in Supplementary Table S3. The interval from transplantation to RARP ranged from approximately 1.5 to 23 years. Both renal grafts were located in the right iliac fossa; both hepatic grafts were orthotopic. All patients were maintained on tacrolimus-based immunosuppression, continued perioperatively at therapeutic trough levels, and renal function remained stable throughout; liver function tests remained within normal limits in both hepatic recipients. Thromboprophylaxis with low-molecular-weight heparin and antibiotic prophylaxis were administered per institutional protocol, and no patient required preoperative bridging of antiplatelet or anticoagulant therapy. Of note, one renal recipient presented with substantially impaired graft function at baseline (creatinine 1.95 mg/dL; eGFR 37 mL/min/1.73 m^2^) in the context of transplant renal artery stenosis, BK virus viraemia and biopsy-proven acute rejection episodes in the past; RARP was nevertheless completed uneventfully in this patient, with stable graft function postoperatively.

## 6. Discussion

This case series describes our initial institutional experience with RARP in four solid organ transplant recipients—two renal and two hepatic—with a particular focus on a novel dual-route ICG fluorescence-guided technique employed in RTRs, and the strategic use of the CMR Versius^®^ robotic platform in a patient with prior orthotopic liver transplantation. To our knowledge, this is the first report describing the intraoperative use of ICG for simultaneous transplanted ureter identification and renal graft vascular mapping during RARP, and the first description of RARP performed using the Versius system in a solid organ transplant recipient.

### 6.1. RARP in Renal Transplant Recipients—Context and Surgical Challenges

The feasibility and safety of RARP in RTRs has been established by several institutional series over the past decade [1,4,5,6,7,22,28,29,31,32,33,34]. RARP has been completed robotically in all reported cases across multicenter series, with no graft injuries recorded; however, postoperative complication rates may be higher than in the non-transplant setting [4,24,25,26,27]. Multicenter data demonstrate no graft loss related to the procedure, with oncological outcomes broadly comparable to the non-transplant setting [4,24,25,26,27]. These data collectively support an active surgical approach in appropriately selected RTRs [1,4,5,25,26,27,43,44], while underscoring the importance of careful perioperative planning.

The principal intraoperative risk unique to RTRs undergoing RARP is injury to the transplanted ureter [4,5]. Unlike the native ureter—which follows a long retroperitoneal course with well-defined anatomical landmarks—the transplanted ureter is shortened, follows an aberrant trajectory from the iliac fossa directly to the bladder dome, and is frequently embedded in dense fibrotic tissue resulting from prior transplant surgery [4,6,7]. A reliable, real-time intraoperative visualization modality is therefore essential in this setting.

A clinically important observation in our series is the universal rate of pathological upstaging: upstaging from clinical to pathological stage occurred in all four patients (100%). Three of four patients (75%) were upstaged to pT3a disease despite preoperative clinical staging of cT2, while the fourth patient was upstaged from cT2b to pT2N0. Although the small sample size precludes definitive conclusions, this finding is consistent with published data in solid organ transplant recipients and may have important implications for clinical decision-making in this population. Shahait et al. [27] and Piana et al. [5] both noted a disproportionately high rate of pT3 disease in RTR cohorts compared to the general RARP population [5,27]. Potential explanations include: the immunosuppression-driven tumour microenvironment promoting local invasiveness [1,2]; potential limitations of multiparametric MRI in the context of post-transplant pelvic fibrosis reducing extracapsular extension detection accuracy; and a selection effect whereby SOTRs present for treatment at a more advanced biologically active stage due to delayed or PSA-confounded screening [3]. These observations suggest that pathological upstaging should be anticipated when counselling solid organ transplant recipients prior to RARP, and that preoperative staging in this population may systematically underestimate the true extent of disease. This has practical implications for patient selection, the decision regarding pelvic lymph node dissection template, and the threshold for adjuvant treatment planning. In our renal transplant recipients, pelvic lymph node dissection was not performed on the ipsilateral side of the graft. The extensive fibrotic reaction and adhesions at the site of the transplanted kidney—particularly around the iliac vessels where the graft is anastomosed—render ipsilateral dissection technically hazardous and associated with a substantial risk of graft vascular injury. Lymphadenectomy was therefore limited to the contralateral side in both RTRs, consistent with published recommendations that ipsilateral templates should be avoided in this setting [4,28,29]. pN0 status was recorded in all patients in whom lymphadenectomy was undertaken; as noted above, in the renal recipients this reflects a contralateral-only dissection and does not exclude graft-side nodal involvement.

### 6.2. ICG-Guided Technique—Novelty and Supporting Evidence

The application of ICG fluorescence for intraoperative ureteral identification has been described across a range of complex pelvic procedures [8,10,11,12]. The first description of intraureteral ICG instillation via a ureteral catheter prior to robotic pelvic surgery demonstrated real-time fluorescent delineation of the ureter throughout the entire procedure, with no adverse effects attributable to ICG administration [11]. Subsequent series in complex robotic gynecological and urological surgery confirmed that ureteral pathways could be identified under NIRF imaging even when not visible under white-light visualization, with no ureteral injuries recorded [8,10,12]. In colorectal robotic surgery, intraureteral ICG achieves precise and prolonged fluorescent visualization compatible with long operative times [10].

Importantly, the pharmacokinetics of ICG are directly relevant to the transplanted ureter context: systemically administered ICG is excreted via the liver, bile ducts, and intestines—not the kidneys—and therefore must be administered by retrograde intraureteral instillation to achieve ureteral fluorescence [11,13]. This property is particularly advantageous in RTRs with reduced renal function, in whom IV-only ICG protocols would fail to produce ureteral signal. Our pre-docking cystoscopic catheterization approach—leaving the ureteral catheter in situ and instilling ICG at the precise moment of surgical need—ensured fluorescent signal availability without interrupting the robotic procedure, representing a practical refinement over techniques requiring intraoperative cystoscopic interruption [11].

The IV bolus component of our dual-route protocol served a distinct and complementary purpose: real-time mapping of the transplanted kidney’s vascular pedicle and qualitative assessment of cortical perfusion. ICG fluorescence videography has been shown to allow high sensitivity and specificity in differentiating between normoperfused and hypoperfused segments of transplanted kidneys during transplant surgery itself, with intraoperative fluorescence angiography shown to predict early graft function [13,45,46]. The extension of this principle to the setting of RARP—where graft perfusion monitoring during a prolonged adjacent surgical procedure has not previously been described—represents a logical and clinically meaningful innovation. To our knowledge, no prior report has described simultaneous dual-route ICG administration (intraureteral + IV) during RARP in an RTR, combining ureteral identification with vascular mapping and graft perfusion monitoring in a single protocol.

### 6.3. RARP in Hepatic Transplant Recipients and the Role of the Versius Platform

Data on RARP in hepatic transplant recipients remain extremely scarce: to our knowledge, only a single case report has been published (Lim et al. [21]), and no series has previously been described [1,5]. Our two hepatic recipients therefore represent, to the best of our knowledge, the largest reported experience in this population, although the evidence base remains far too small to support generalisable conclusions. The challenges in this population differ fundamentally from RTRs: while native ureteral anatomy is preserved, the prior laparotomy—typically a chevron (bilateral subcostal) incision for orthotopic liver transplantation—creates extensive abdominal wall scarring, adhesion burden, and altered fascial compliance that complicates trocar placement and peritoneal access. A further hazard specific to this population, illustrated by our case, is the risk of abdominal wall bleeding at trocar insertion sites. Prior hepatic transplantation and underlying hepatic vascular disease may result in enhanced collateral circulation within the abdominal wall; in our patient, the source of postoperative bleeding requiring embolization was identified at the assistant trocar insertion site, most likely arising from such collateral vessels. This risk should be anticipated preoperatively through careful review of cross-sectional imaging for abdominal wall vascularity, with particular attention to the planned trocar sites. Critically, given that ureteral anatomy is preserved in this group, neither ICG nor Firefly^®^ NIRF imaging was employed in our hepatic transplant recipients. The fluorescence-guided protocol was reserved exclusively for RTRs, where the aberrant transplanted ureter and adjacent graft vasculature represent a specific, well-defined intraoperative hazard not present in hepatic transplant recipients. The hepatic recipient who bled merits detailed consideration. Preoperative coagulation was essentially normal (INR 1.09; APTT 32 s; prothrombin index 93%), but the platelet count was reduced at 119 × 10^9^/L, consistent with portal hypertension following previous endoscopic banding of oesophageal varices; the postoperative nadir was 76 × 10^9^/L. Bleeding arose from a small branch of the anterior trunk of the left internal iliac artery, producing an extensive left pelvic haematoma, and was managed by selective transarterial embolisation (Progreat 2.7F microcatheter; Glubran/Lipiodol 1:5) with transfusion of 2.5 units of packed red cells (Clavien–Dindo IIIa); follow-up imaging confirmed no active bleeding and the patient recovered fully. Importantly, the preoperative CT was not specifically reviewed for abdominal-wall or pelvic venous collaterals, and such collaterals were not described in the standard radiology report. Given the portal hypertension present in this recipient, we regard this as a lesson learned: dedicated preoperative mapping of venous collaterals should be considered in hepatic transplant recipients, both to inform trocar placement and to anticipate bleeding risk.

In one of our hepatic transplant recipients, the CMR Versius^®^ system was used for RARP. The Versius platform’s modular architecture and independent arm docking allow trocar placement to be tailored to individual patient anatomy without the spatial constraints imposed by a single integrated cart system [47]. Building on our institutional experience with the Versius system for standard RARP—including a consecutive series of 58 cases [17]—infra-umbilical port placement was employed to entirely avoid the transplant scar, enabling safe peritoneal access and unobstructed instrument triangulation for pelvic dissection. This application of the Versius platform in a transplant recipient has not been previously described and highlights the potential role of platform selection—not merely surgical technique—as a component of operative planning in complex cases. This single-case experience is descriptive and does not, in itself, demonstrate any advantage of the Versius platform over established robotic systems; comparative studies would be required to substantiate any such claim. Comparative evidence in this population was, until recently, entirely absent. Slota et al. [36] have now reported the first propensity-matched analysis of RARP in kidney transplant recipients, matching eight recipients 1:4 against non-transplant controls. Despite a substantially higher comorbidity burden, recipients had comparable operative times, blood loss and length of stay, with no graft injuries or acute kidney injury, and neither positive surgical margin nor biochemical recurrence rates differed significantly from controls; allograft function remained stable at 12 months. A higher 30-day urinary tract infection rate was the single outcome that differed, plausibly reflecting immunosuppression and prolonged catheterisation. These findings are consistent with our own perioperative observations and support the position that transplant status alone should not preclude a curative surgical approach. Notably, however, no comparative study—including that of Slota et al. [36]—has examined fluorescence guidance in this setting, and the incremental value of ICG therefore remains untested against a non-ICG comparator.

### 6.4. Limitations

This series is limited by its small sample size, retrospective design, and single-institution experience. Graft function was preserved in all patients with no graft dysfunction observed, consistent with published multicenter data showing stable renal function at one year after RARP in RTRs [4,24,25,26]. Uninterrupted immunosuppression throughout the perioperative period is essential and was maintained in all cases. The heterogeneity of transplant type and robotic platform precludes direct comparative analysis. ICG was not protocolized across all cases, reflecting a pragmatic anatomy-driven approach. A technical limitation inherent to the da Vinci Firefly^®^ system is that active surgical dissection cannot be performed while in NIRF mode: the system requires the surgeon to toggle between white-light and fluorescence imaging, temporarily interrupting dissection at each anatomical verification step. This represents a practical workflow constraint, particularly during critical dissection phases where continuous fluorescence guidance would be most beneficial. Emerging laparoscopic imaging platforms—such as the Karl Storz Image1 S™ Rubina^®^ system—offer a simultaneous white-light and NIRF overlay mode, enabling real-time fluorescence visualization without interrupting surgical activity. Whether comparable overlay capability will become available in future robotic platforms remains to be seen, but would represent a clinically meaningful technical advance for procedures such as RARP in renal transplant recipients. An alternative fluorophore for intraoperative ureteral identification is methylene blue (MB), which unlike ICG undergoes partial renal excretion and can therefore be administered intravenously to achieve ureteral fluorescence under NIR imaging—an approach previously described and reviewed in the context of urological applications [48]. The combined use of ICG and MB in multispectral fluorescence imaging has been explored in colorectal surgery, demonstrating complementary near-infrared signal characteristics of potential relevance to complex pelvic procedures [49]. However, MB-based NIR ureteral identification has not yet been implemented in robotic surgical platforms, and its applicability in the transplant setting—where renal function may be reduced—would require further evaluation. Longer-term oncological outcomes—biochemical recurrence-free survival, continence recovery, and graft survival—require extended follow-up beyond the scope of this initial report. Prospective multicenter registries are needed to standardize RARP technique and ICG protocols in this population. The median follow-up of 12 months (range 4–39) remains short for assessing oncological (biochemical recurrence-free survival) and functional endpoints; accordingly, our conclusions are restricted to perioperative feasibility and early outcomes, and no inference is made regarding long-term cancer control or functional recovery. This study also lacks a non-ICG comparator arm and was not designed to determine whether ICG guidance measurably improves complication rates, operative safety, or graft preservation; the value of ICG demonstrated here is qualitative—real-time intraoperative navigation—and any effect on measurable outcomes remains to be established in controlled studies.

## 7. Conclusions

This case series demonstrates that a dual-route ICG fluorescence protocol—pre-docking intraureteral catheterization combined with IV bolus at the same operative stage—enables real-time identification of the transplanted ureter and graft vascular mapping during RARP in renal transplant recipients, with no ureteral injuries or graft-related complications. To our knowledge, this is the first description of this combined approach. In one hepatic transplant recipient, the CMR Versius^®^ system’s flexible port configuration enabled fully infra-umbilical trocar placement, entirely avoiding the chevron transplant scar—a configuration not previously described in a transplant recipient.

Graft function was preserved in all patients, with no evidence of graft dysfunction during follow-up. These results suggest that RARP is technically feasible in carefully selected solid organ transplant recipients when performed at experienced robotic centres with dedicated multidisciplinary transplant support; they do not, however, establish its safety or oncological efficacy, which require confirmation in larger studies. Larger prospective multicenter series are needed to validate ICG protocols and define long-term outcomes in this population. These observations are preliminary and hypothesis-generating; given the small sample size, the heterogeneity of transplant types, and the short follow-up, they should be interpreted with caution. Conclusions regarding hepatic transplant recipients rest on only two patients and cannot be generalised, and the single-case Versius^®^ experience should be regarded as a technical observation that does not, by itself, establish any advantage over established robotic platforms.

## Figures and Tables

**Table 1:** Patient demographics, transplant characteristics, and preoperative oncological data.

Parameter	Patient 1	Patient 2	Patient 3	Patient 4
Transplant type	Kidney	Kidney	Liver	Liver
Robotic platform	da Vinci Xi	da Vinci Xi	da Vinci Xi	CMR Versius^®^
Age (years)	64	59	49	60
BMI (kg/m^2^)	27.1	31.0	32.0	29.0
PSA preoperative (ng/mL)	9.96	5.33 *	10.3	10.8
Prostate volume (mL)	32	49	27	30
Biopsy Gleason/ISUP GG	4 + 3/ISUP GG3	4 + 3/ISUP GG3	3 + 4/ISUP GG2	4 + 3/ISUP GG3
Positive cores	3/7	6/8	6/8	6/11
PI-RADS	4	5	4	4
Clinical stage	cT2a	cT2b	cT2b	cT2a
Suspicious ECE (MRI)	No	No	No	No
Suspicious SVI (MRI)	No	No	No	No
ICG used	Yes (dual-route)	Yes (dual-route)	No	No
Immunosuppression	+	+	+	+

* PSA measured under tacrolimus (calcineurin inhibitor) immunosuppression. Unlike mTOR inhibitors (e.g., sirolimus or everolimus), tacrolimus does not suppress PSA levels; this value is not subject to pharmacological PSA confounding. ICG = indocyanine green; ISUP GG = ISUP Grade Group; ECE = extracapsular extension; SVI = seminal vesicle invasion.

**Table 2:** Operative, pathological, and follow-up outcomes.

Parameter	Patient 1 (Kidney)	Patient 2 (Kidney)	Patient 3 (Liver)	Patient 4 (Liver/Versius)
Robotic platform	da Vinci Xi	da Vinci Xi	da Vinci Xi	CMR Versius^®^
Operative time (min)	206	182	169	150
Console time (min)	165	155	145	135
EBL (mL)	400	300	800	200
Conversion to open	No	No	No	No
Catheterization (days)	7	7	7	11
Final Gleason/ISUP GG	4 + 3/ISUP GG3	3 + 4/ISUP GG2	3 + 4/ISUP GG2	4 + 3/ISUP GG3
pTNM	pT3aN0	pT3aN0	pT2N0	pT3aN0
Upstaging TNM	Yes	Yes	No	Yes
Upstaging/Downstaging Gleason	Concordant	Downgrading	Concordant	Upgrading
Surgical margins	R0	R0	R0	R0
Hospital stay (days)	3	3	6	4
Complications (C-D)	None	None	IIIa (embolization)	None
Anastomotic stricture	No	No	No	No
Urinary incontinence 3M	0 pads/day	Stress (mild)	Stress (mild)	Mild
PSA at 3 months (ng/mL)	0.010	0.003	0.007	0.020
Adjuvant/salvage radio-/hormonotherapy	No	No	No	Yes (salvage RT + ADT)

EBL = estimated blood loss; C-D = Clavien-Dindo classification; PSA = prostate-specific antigen; R0 = negative surgical margins.

**Table 3:** Summary of included studies: RARP in solid organ transplant recipients.

Author (Year)	*n*	Transplant	Platform	Op Time (min)	EBL (mL)	PSM (n/N)	Graft Injury	ICG	Key Finding
Marra et al. 2022 [4]	41	Kidney	da Vinci	201	NR	7/41	0/41	No	Largest RARP RTR series; no graft injury
Schmidt et al. 2024 [24]	12 *	Kidney	da Vinci	NR	NR	3/12	0/12	No	9-centre; RARP vs. open compared
Felber et al. 2020 [25]	39	Kidney	da Vinci	180	150	5/39 *	0/39	No	5-centre French; matched controls
Léonard et al. 2020 [26]	12	Kidney	da Vinci	NR	NR	NR	0/12	No	Multicenter comparative study
Shahait et al. 2021 [27]	14	Kidney	da Vinci	130	110	3/14	0/14	No	Largest single-centre series
Mistretta et al. 2019 [6]	9	Kidney	da Vinci	195	170	2/9	0/9	No	Standard + Retzius-sparing
Sirisopana et al. 2021 [7]	5	Kidney	Mixed	218	NR	2/5	0/5	No	First Thai series
Polcari et al. 2012 [28]	7	Kidney	da Vinci	186	NR	2/7	0/7	No	First US multicentre
Wagener et al. 2012 [29]	4	Kidney	da Vinci	NR	NR	1/4	0/4	No	Extraperitoneal approach
Le Clerc et al. 2015 [30]	12	Kidney	da Vinci	241	NR	4/12 *	0/12	No	French series (12 consecutive)
Moreno Sierra et al. 2016 [31]	4	Kidney	da Vinci	NR	NR	NR	0/4	No	Spanish series + review
Smith et al. 2011 [32]	3	Kidney	da Vinci	NR	NR	NR	0/3	No	Early RTR series; port modified to avoid allograft
Jenjitranant et al. 2016 [33]	1	Kidney	da Vinci	NR	NR	0/1	0/1	No	Retzius-sparing; first Thailand
Ghazi et al. 2012 [34]	3	Kidney	da Vinci	NR	NR	NR	0/3	No	Extraperitoneal modified
Minami et al. 2020 [23]	1	Kidney	da Vinci	NR	NR	0/1	0/1	No	Second transplant recipient
Jhaveri et al. 2008 [22]	1	Kidney	da Vinci	NR	NR	0/1	0/1	No	First RARP in RTR ever
Leow et al. 2025 [35]	4	Kidney	da Vinci	NR	NR	1/4	0/4	No	Retzius-sparing; no 30-day complications; eGFR unchanged (*p* = 0.37)
Slota et al. 2025 [36]	8	Kidney	da Vinci	198.5	125	2/8	0/8	No	Propensity-matched 1:4 vs. non-KTR; only comparative study
Waeckel et al. 2026 [37]	8	Kidney	Robotic	150	50	NS	0/8	No	Open (*n* = 11) vs. robotic (*n* = 8); EBL 50 vs. 500 mL (*p* < 0.001)
Iizuka et al. 2016 [38]	3	Kidney	da Vinci	162	52	0/3	0/3	No	Asian RTR series; ports moved medially; 1/3 BCR treated with ADT [overlapping institutional cohort: Tokyo Women’s Medical Univ.]
Fukuda et al. 2025 [39]	1	Kidney	Hugo RAS	NR	NR	NR	0/1	No	First RARP using Hugo platform in a transplant recipient
Fang et al. 2018 [40]	1	Kidney	da Vinci	NR	NR	NR	0/1	No	First RARP in a transplant recipient in China
Lim et al. 2019 [21]	1	Liver	da Vinci	NR	NR	NR	0/1	No	Only prior report of RARP in a hepatic transplant recipient
Present series	4	Kidney + Liver	da Vinci Xi/Versius	176	350	0/4	0/4	Yes	First ICG dual-route + Versius in SOTRs

* Value derived from the reported percentage where the primary study did not state the absolute count. Positive surgical margins are reported as the number of patients with a positive margin over the total (n/N); percentages in the original publications that did not correspond to whole patient numbers have been converted accordingly. The Iizuka et al. [38] cohort originates from the same institution (Tokyo Women’s Medical University) as two reports excluded for cohort overlap (Iwamoto et al. 2018 [41]; Kobari et al. 2022 [42]); only the most complete report was retained. The present four-patient series is listed separately in the final row and is not included in the pooled literature denominator of 194 patients from the 23 published studies. NR = not reported; PSM = positive surgical margin; EBL = estimated blood loss; ICG = indocyanine green; NS = not significant. Two secondary systematic reviews (Piana et al. 2023 [5]; Zeng et al. 2018 [20]) were consulted for contextual reference but are not listed as primary studies, as their patient populations overlap with the included primary series.

## Data Availability

The data presented in this study are available on request from the corresponding author. The data are not publicly available due to privacy and ethical restrictions related to patient confidentiality.

## Supplementary Materials

The following supporting information can be downloaded at: https://www.mdpi.com/article/10.3390/cancers18152408/s1, Table S1: PRISMA 2020 checklist; Table S2: JBI critical appraisal (methodological quality/risk of bias) of the included studies; Table S3: per-patient transplant-specific, perioperative, pathological and follow-up data; Table S4: database-specific search strategies.
