# Supplementary material for: Robot-Assisted Radical Prostatectomy in Solid Organ Transplant Recipients: Initial Experience and Systematic Review
Source: Cancers (Basel). 2026 Jul 26;18(15):2408. doi: 10.3390/cancers18152408 (PMC13464858; doi:10.3390/cancers18152408)
Supplement: Supplementary file 1 [file cancers-18-02408-s001.zip › cancers-4412328-supplementary.pdf]

# Supplementary Materials

Manuscript cancers-4412328 — Robot-Assisted Radical Prostatectomy in Solid Organ Transplant Recipients: Initial Experience and Systematic Review

*Note: Table S1 (PRISMA 2020 checklist).*

## PRISMA 2020 Checklist

*Robot-Assisted Radical Prostatectomy in Solid Organ Transplant Recipients: Initial Experience and Systematic Review*

Section/page references correspond to the revised manuscript. The systematic review component used a qualitative (narrative) synthesis. A formal methodological quality / risk-of-bias appraisal was performed using the JBI Critical Appraisal Checklists (Table S2). Items relating specifically to meta-analysis and to GRADE certainty-of-evidence scoring are marked "Not applicable" with the reason given, as neither a meta-analysis nor a GRADE assessment was undertaken.

| Section and Topic | Item # | Checklist item | Location where item is reported |
|---|---|---|---|
| **TITLE** | | | |
| Title | 1 | Identify the report as a systematic review. | Title; Abstract |
| **ABSTRACT** | | | |
| Abstract | 2 | See the PRISMA 2020 for Abstracts checklist. | Abstract |
| **INTRODUCTION** | | | |
| Rationale | 3 | Describe the rationale for the review in the context of existing knowledge. | Section 1 (Introduction) |
| Objectives | 4 | Provide an explicit statement of the objective(s) or question(s) the review addresses. | Section 1 (final paragraph); Section 2.1 |
| **METHODS** | | | |
| Eligibility criteria | 5 | Specify the inclusion and exclusion criteria for the review and how studies were grouped for the syntheses. | Section 2.2 |
| Information sources | 6 | Specify all databases, registers, websites, organisations, reference lists and other sources searched or consulted to identify studies. Specify the date when each source was last searched or consulted. | Section 2.2 (PubMed/MEDLINE and Web of Science Core Collection; reference lists / citation searching; last searched 13 July 2026). Full strategies in Table S4. |
| Search strategy | 7 | Present the full search strategies for all databases, registers and websites, including any filters and limits used. | Section 2.2 (full Boolean string) |
| Selection process | 8 | Specify the methods used to decide whether a study met the inclusion criteria of the review, including how many reviewers screened each record and each report retrieved, whether they worked independently, and if applicable, details of automation tools used in the process. | Section 2.2 (two reviewers, independent screening, consensus) |
| Data collection process | 9 | Specify the methods used to collect data from reports, including how many reviewers collected data from each report, whether they worked independently, any processes for obtaining or confirming data from study investigators, and if applicable, details of automation tools used in the process. | Section 2.2; Section 5.1 (data extracted into Table 3 by two reviewers) |
| Data items | 10a | List and define all outcomes for which data were sought. Specify whether all results that were compatible with each outcome domain in each study were sought, and if not, the methods used to decide which results to collect. | Section 2.2; Table 3 (operative time, EBL, PSM, graft injury, ICG use) |
| Data items | 10b | List and define all other variables for which data were sought. Describe any assumptions made about any missing or unclear information. | Table 3 (n, transplant type, platform); missing values marked NR |
| Study risk of bias assessment | 11 | Specify the methods used to assess risk of bias in the included studies, including details of the tool(s) used, how many reviewers assessed each study and whether they worked independently, and if applicable, details of automation tools used in the process. | Two reviewers independently appraised each study using the JBI Critical Appraisal Checklists for Case Series, Case Reports and Cohort Studies, with disagreements resolved by consensus (Section |

| Section and Topic | Item # | Checklist item | Location where item is reported |
|---|---|---|---|
| | | | 2.2; Table S2). No formal tool applied (Section 6.4) |
| Effect measures | 12 | Specify for each outcome the effect measure(s) (e.g. risk ratio, mean difference) used in the synthesis or presentation of results. | Not applicable — descriptive synthesis only; no pooled effect measures |
| Synthesis methods | 13a | Describe the processes used to decide which studies were eligible for each synthesis. | Section 2.2; Section 5.1 |
| Synthesis methods | 13b | Describe any methods required to prepare the data for presentation or synthesis, such as handling of missing summary statistics, or data conversions. | Not applicable — no data conversion; missing values reported as NR (Table 3) |
| Synthesis methods | 13c | Describe any methods used to tabulate or visually display results of individual studies and syntheses. | Section 5.2; Table 3 |
| Synthesis methods | 13d | Describe any methods used to synthesize results and provide a rationale for the choice(s). If meta-analysis was performed, describe the model(s), method(s) to identify the presence and extent of statistical heterogeneity, and software package(s) used. | Section 5.2 (qualitative/narrative synthesis; ranges reported). No meta-analysis performed, given heterogeneity and small samples |
| Synthesis methods | 13e | Describe any methods used to explore possible causes of heterogeneity among study results (e.g. subgroup analysis, meta-regression). | Not applicable — no meta-analysis |
| Synthesis methods | 13f | Describe any sensitivity analyses conducted to assess robustness of the synthesized results. | Not applicable — no meta-analysis |
| Reporting bias assessment | 14 | Describe any methods used to assess risk of bias due to missing results in a synthesis (arising from reporting biases). | Not applicable — narrative synthesis |
| Certainty assessment | 15 | Describe any methods used to assess certainty (or confidence) in the body of evidence for an outcome. | Not applicable — GRADE not applied |
| **RESULTS** | | | |
| Study selection | 16a | Describe the results of the search and selection process, from the number of records identified in the search to the number of studies included in the review, ideally using a flow diagram. | Section 5.1; Figure 1 (PRISMA flow diagram) |
| Study selection | 16b | Cite studies that might appear to meet the inclusion criteria, but which were excluded, and explain why they were excluded. | Section 5.1 (14 excluded at title/abstract screening and 9 at full-text review, with reasons); Figure 1 |
| Study characteristics | 17 | Cite each included study and present its characteristics. | Section 5.2; Table 3 |
| Risk of bias in studies | 18 | Present assessments of risk of bias for each included study. | Risk-of-bias appraisal for every included study is presented in Table S2 (Section 5.1) |
| Results of individual studies | 19 | For all outcomes, present, for each study: (a) summary statistics for each group (where appropriate) and (b) an effect estimate and its precision (e.g. confidence/credible interval), ideally using structured tables or plots. | Table 3 (per-study outcomes) |
| Results of syntheses | 20a | For each synthesis, briefly summarise the characteristics and risk of bias among contributing studies. | Section 5.2 |
| Results of syntheses | 20b | Present results of all statistical syntheses conducted. If meta-analysis was done, present for each the summary estimate and its precision (e.g. confidence/credible interval) and measures of statistical heterogeneity. If comparing groups, describe the direction of the effect. | Section 5.2 (ranges across studies). Not applicable — no meta-analysis |
| Results of syntheses | 20c | Present results of all investigations of possible causes of heterogeneity among study results. | Not applicable — no meta-analysis |
| Results of syntheses | 20d | Present results of all sensitivity analyses conducted to assess the robustness of the synthesized results. | Not applicable — no meta-analysis |
| Reporting biases | 21 | Present assessments of risk of bias due to missing results (arising from reporting biases) for each synthesis assessed. | Not applicable — narrative synthesis |

| Section and Topic | Item # | Checklist item | Location where item is reported |
|---|---|---|---|
| Certainty of evidence | 22 | Present assessments of certainty (or confidence) in the body of evidence for each outcome assessed. | Not applicable — GRADE not applied |
| **DISCUSSION** | | | |
| Discussion | 23a | Provide a general interpretation of the results in the context of other evidence. | Section 6 (Discussion) |
| Discussion | 23b | Discuss any limitations of the evidence included in the review. | Section 6.4 |
| Discussion | 23c | Discuss any limitations of the review processes used. | Section 6.4 |
| Discussion | 23d | Discuss implications of the results for practice, policy, and future research. | Section 6.4; Section 7 (Conclusions) |
| **OTHER INFORMATION** | | | |
| Registration and protocol | 24a | Provide registration information for the review, including the register name and registration number, or state that the review was not registered. | The systematic review was not registered (state in Methods/back matter) |
| Registration and protocol | 24b | Indicate where the review protocol can be accessed, or state that a protocol was not prepared. | No separate protocol was prepared |
| Registration and protocol | 24c | Describe and explain any amendments to information provided at registration or in the protocol. | Not applicable |
| Support | 25 | Describe sources of financial or non-financial support for the review, and the role of the funders or sponsors in the review. | Funding section (back matter) |
| Competing interests | 26 | Declare any competing interests of review authors. | Conflicts of Interest section (back matter) |
| Availability of data, code and other materials | 27 | Report which of the following are publicly available and where they can be found: template data collection forms; data extracted from included studies; data used for all analyses; analytic code; any other materials used in the review. | Data Availability Statement (back matter); extracted data in Table 3 |

*Reference: Page MJ, McKenzie JE, Bossuyt PM, et al. The PRISMA 2020 statement: an updated guideline for reporting systematic reviews. BMJ. 2021;372:n71. (Manuscript reference [14].)*

## Supplementary Materials

Manuscript cancers-4412328 — Robot-Assisted Radical Prostatectomy in Solid Organ Transplant Recipients: Initial Experience and Systematic Review

*Note: Table S1 (PRISMA 2020 checklist) is provided above; Tables S2–S4 follow.*

## Table S2. Methodological quality / risk-of-bias appraisal (JBI Critical Appraisal Checklists)

Case series were appraised with the JBI Critical Appraisal Checklist for Case Series (items 1–10); case reports with the JBI Checklist for Case Reports (items 1–8; items 9–10 not applicable); and the single matched cohort study (Slota et al.) with the JBI Checklist for Cohort Studies. Two reviewers appraised each study independently, with disagreements resolved by consensus.

*Y = yes; N = no; U = unclear; NA = not applicable.*

| Study (Year) | Design | 1 | 2 | 3 | 4 | 5 | 6 | 7 | 8 | 9 | 10 | Overall |
|---|---|---|---|---|---|---|---|---|---|---|---|---|
| Marra et al. 2022 [4] | Multicentre case series | Y | Y | Y | Y | Y | Y | Y | Y | Y | Y | Low–moderate |
| Schmidt et al. 2024 [24] | Multicentre case series | Y | Y | Y | Y | U | Y | Y | Y | Y | Y | Moderate |
| Felber et al. 2020 [25] | Multicentre case series | Y | Y | Y | Y | Y | Y | Y | Y | Y | Y | Low–moderate |
| Léonard et al. 2020 [26] | Multicentre case series | Y | Y | Y | U | U | Y | U | U | Y | U | Moderate |
| Shahait et al. 2021 [27] | Single-centre case series | Y | Y | Y | Y | Y | Y | Y | Y | Y | U | Moderate |
| Mistretta et al. 2019 [6] | Single-centre case series | Y | Y | Y | Y | Y | Y | Y | Y | Y | U | Moderate |
| Sirisopana et al. 2021 [7] | Single-centre case series | Y | Y | Y | U | U | Y | Y | U | Y | N | Moderate–high |
| Polcari et al. 2012 [28] | Multicentre case series | Y | Y | Y | U | U | Y | Y | U | Y | N | Moderate–high |
| Wagener et al. 2012 [29] | Single-centre case series | Y | Y | Y | U | U | Y | U | U | U | N | High |
| Le Clerc et al. 2015 [30] | Single-centre case series | Y | Y | Y | Y | Y | Y | Y | U | Y | N | Moderate |
| Moreno Sierra et al. 2016 [31] | Case series + review | Y | Y | U | U | U | Y | U | U | U | N | High |
| Smith et al. 2011 [32] | Single-centre case series | Y | Y | U | U | U | Y | U | U | U | N | High |
| Ghazi et al. 2012 [34] | Single-centre case series | Y | Y | U | U | U | Y | U | U | U | N | High |
| **Case reports (JBI Critical Appraisal Checklist for Case Reports; items 9–10 not applicable)** | | | | | | | | | | | | |
| Jenjitranant et al. 2016 [33] | Case report | Y | Y | Y | Y | Y | Y | Y | Y | NA | NA | Moderate |
| Minami et al. 2020 [23] | Case report | Y | Y | Y | Y | Y | Y | Y | Y | NA | NA | Moderate |
| Jhaveri et al. 2008 [22] | Case report | Y | Y | Y | Y | Y | Y | U | Y | NA | NA | Moderate |
| **Studies added in the updated search (13 July 2026)** | | | | | | | | | | | | |
| Leow et al. 2025 [35] (n=4) | Single-centre case series | Y | Y | Y | Y | Y | Y | Y | Y | Y | U | Moderate |
| Slota et al. 2025 [36] (n=8) | Matched | Y | Y | Y | Y | Y | Y | Y | Y | Y | Y | Low |

| | | | | | | | | | | | | |
|---|---|---|---|---|---|---|---|---|---|---|---|---|
| | cohort (JBI cohort tool) | | | | | | | | | | | |
| Waeckel et al. 2026 [37] (n=8) | Comparative cohort (JBI cohort tool) | Y | Y | Y | Y | Y | Y | Y | Y | Y | Y | Low–moderate |
| Iizuka et al. 2016 [38] (n=3) | Single-centre case series | Y | Y | Y | Y | Y | Y | Y | Y | Y | U | Moderate |
| Fukuda et al. 2025 [39] (n=1) | Case report | Y | Y | Y | Y | Y | Y | Y | Y | NA | NA | Moderate |
| Fang et al. 2018 [40] (n=1) | Case report | Y | Y | Y | Y | Y | Y | Y | Y | NA | NA | Moderate |
| Lim et al. 2019 [21] (n=1, liver) | Case report | Y | Y | Y | Y | Y | Y | U | Y | NA | NA | Moderate |
| **Present series (2026)** | **Single-centre case series** | Y | Y | Y | Y | Y | Y | Y | Y | Y | U | **Moderate** |

JBI Case Series items: (1) clear inclusion criteria; (2) condition measured in a standard, reliable way; (3) valid identification methods; (4) consecutive inclusion; (5) complete inclusion; (6) demographics reported; (7) clinical information reported; (8) outcomes/follow-up reported; (9) site demographics reported; (10) appropriate statistical analysis.

Summary: across the 23 included primary studies (194 patients), the evidence base consists predominantly of retrospective, single-arm case series and case reports, most of which do not report consecutive or complete enrolment and which lack a comparator arm. Only two studies provide comparative data — the propensity-matched cohort of Slota et al. (2025) and the open-versus-robotic comparison of Waeckel et al. (2026) — and both were appraised using the JBI checklist for cohort studies. The overall certainty of evidence remains low.

*Appraisals were performed independently by two reviewers against the full text of each included study, with disagreements resolved by consensus.*

## Table S3. Per-patient transplant-specific, perioperative, pathological and outcome data

*RTR = renal transplant recipient; LTR = liver transplant recipient; PLND = pelvic lymph node dissection; LVI = lymphovascular invasion; PNI = perineural invasion; PRBC = packed red blood cells; RT = radiotherapy; ADT = androgen deprivation therapy; LFT = liver function test.*

| Variable | RTR1 | RTR2 | LTR1 | LTR2 |
|---|---|---|---|---|
| Transplanted organ | Kidney | Kidney | Liver (orthotopic) | Liver (orthotopic) |
| Age at RARP (years) | 64 | 59 | 49 | 60 |
| Interval transplant → RARP | 11.6 years | 3.3 years | 1.5 years | ≈23 years |
| Graft location | Right iliac fossa | Right iliac fossa | Orthotopic | Orthotopic |
| Relevant graft history | Chronic glomerulonephritis of native kidneys; proteinuria | Transplant renal artery stenosis; BK viraemia; prior biopsy-proven acute rejection; impaired graft function | Alcohol-related cirrhosis; portal hypertension (prior variceal banding) | None reported |
| Immunosuppression | Tacrolimus-based | Tacrolimus-based | Tacrolimus-based | Tacrolimus-based |
| Baseline creatinine (mg/dL) / eGFR | 1.19 / 64 | 1.95 / 37 | 0.81 / >90 | 0.85 / >90 |
| Postoperative graft function | Stable | Stable | Stable; LFTs within normal limits | Stable; LFTs within normal limits |
| Preoperative PSA (ng/mL) | 9.96 | 5.33 | 10.36 | 10.8 |
| Biopsy Gleason / ISUP GG | 4+3 (GG3) | 4+3 (GG3) | 3+4 (GG2) | 3+4 (GG2) |
| Preoperative clinical stage (cT) | cT2a | cT2b | cT2b | cT2a |
| Robotic platform | da Vinci | da Vinci | da Vinci | Versius® |
| Dual-route ICG | Yes | Yes | No | No |
| Ureteral catheter (ICG route) | 5 Fr open-ended; removed intraoperatively | 5 Fr open-ended; removed intraoperatively | N/A | N/A |
| Operative modification | Contralateral PLND only; ICG ureteric tracking | Adhesiolysis graft–bladder; contralateral PLND only; ICG | Standard transperitoneal | Infra-umbilical ports placed lower to avoid chevron scar |

| | | | | |
|---|---|---|---|---|
| Estimated blood loss (mL) | 400 | 300 | 800 | 200 |
| PLND template / laterality | Contralateral (left) only | Contralateral (left) only | Bilateral obturator | Bilateral obturator |
| Nodes removed / positive | 2 / 0 | 2 / 0 | 2 / 0 | 1 / 0 |
| Pathological stage | pT3a N0 | pT3a N0 | pT2 N0 | pT3a N0 |
| Specimen Gleason / ISUP GG | 4+3 (GG3) | 3+4 (GG2) | 3+4 (GG2) | 4+3 (GG3) |
| Surgical margins | Negative (R0) | Negative (R0) | Negative (R0) | Negative (R0) |
| LVI / PNI | Present / Present | Absent / Present | Present / Present | Not reported / Present |
| Complication (Clavien–Dindo) | None | None | Pelvic haemorrhage, IIIa (embolisation; 2.5 U PRBC) | None |
| Graft injury | None | None | None | None |
| Nerve-sparing (based on preoperative cT) | Bilateral (cT2a) | Bilateral (cT2b) | Bilateral (cT2b) | Bilateral (cT2a) |
| Continence (pad use) | Pad-free | Pad-free | Pad-free | Pad-free |
| Follow-up duration (months) | 16 | 8 | 4 | 39 |
| Last PSA (ng/mL) | 0.006 | 0.003 | 0.007 | 0.02 (post-salvage) |
| Biochemical recurrence | No | No | No | Yes — at 4 months (PSA 0.23); salvage RT to prostate bed + ADT per EAU schedule |
| Erectile function (IIEF) | Not assessed | Not assessed | Not assessed | Not assessed |

Note on PLND: in both renal transplant recipients the graft lay in the right iliac fossa and dissection was deliberately restricted to the contralateral (left) side to protect the transplant vasculature and ureter. pN0 status in these patients therefore reflects a contralateral-only dissection and does not exclude graft-side nodal involvement.

## Table S4. Database-specific search strategies

Searches were last updated on 13 July 2026. No language or date restrictions were applied. The review was not prospectively registered. The same search string was applied across both databases; six additional studies were identified through citation searching of reference lists.

| Database | Search string | Records |
|---|---|---|
| PubMed / MEDLINE | ("robot-assisted radical prostatectomy" OR "RARP" OR "robotic prostatectomy" OR "laparoscopic radical prostatectomy") AND ("renal transplant" OR "kidney transplant" OR "liver transplant" OR "hepatic transplant" OR "solid organ transplant") AND ("prostate cancer" OR "prostate neoplasm") | 36 |
| Web of Science Core Collection | TS=(("robot-assisted radical prostatectomy" OR "RARP" OR "robotic prostatectomy" OR "laparoscopic radical prostatectomy") AND ("renal transplant" OR "kidney transplant" OR "liver transplant" OR "hepatic transplant" OR "solid organ transplant") AND ("prostate cancer" OR "prostate neoplasm")) | 35 |
| Citation searching | Reference lists of all included articles and of relevant systematic reviews (Piana et al.; Zeng et al.) were screened manually for additional eligible studies. | 6 |

*A total of 71 records were retrieved from the two databases; after removal of 29 duplicates, 42 unique records were screened. Six further studies were identified through citation searching. The full selection process is presented in Figure 1 (PRISMA 2020 flow diagram) in the main manuscript.*
